# Transipedia.org: k-mer-based exploration of large RNA sequencing datasets and application to cancer data

**DOI:** 10.1186/s13059-024-03413-5

**Published:** 2024-10-10

**Authors:** Chloé Bessière, Haoliang Xue, Benoit Guibert, Anthony Boureux, Florence Rufflé, Julien Viot, Rayan Chikhi, Mikaël Salson, Camille Marchet, Thérèse Commes, Daniel Gautheret

**Affiliations:** 1 IRMB, INSERM U1183, Hopital Saint-Eloi, Universite de Montpellier, Montpellier, France; 2 CRCT, Inserm, CNRS, Université Toulouse III-Paul Sabatier, Centre de Recherches en Cancérologie de Toulouse, Toulouse, France; 3 I2BC, Université Paris-Saclay, CNRS, CEA, Gif sur Yvette, France; 4 Department of Medical Oncology, Biotechnology and Immuno-Oncology Platform, University Hospital of Besançon, Besançon, France; 5 INSERM, EFS BFC, UMR1098, RIGHT, University of Franche-Comté, Interactions Greffon-Hôte-Tumeur/Ingénierie Cellulaire et Génique, Besançon, France; 6 Institut Pasteur, Université Paris Cité, Paris, France; 7 Université de Lille, CNRS, Centrale Lille, UMR 9189 CRIStAL, F-59000 Lille, France

**Keywords:** RNA-seq, Transcriptomics, Bioinformatics, RNA-processing, Non-coding RNA

## Abstract

Indexing techniques relying on k-mers have proven effective in searching for RNA sequences across thousands of RNA-seq libraries, but without enabling direct RNA quantification. We show here that arbitrary RNA sequences can be quantified in seconds through their decomposition into k-mers, with a precision akin to that of conventional RNA quantification methods. Using an index of the Cancer Cell Line Encyclopedia (CCLE) collection consisting of 1019 RNA-seq samples, we show that k-mer indexing offers a powerful means to reveal non-reference sequences, and variant RNAs induced by specific gene alterations, for instance in splicing factors.

## Introduction

With the generalization of RNA-sequencing (RNA-seq) analysis in most areas of biology and medicine, RNA-seq repositories have grown in size to millions of samples. The Sequence Read Archive (SRA) alone contains 1.8 million public human RNA-sequencing experiments as of January 2024. Due to high costs of RNA-seq data download and reanalysis, exploration of RNA-seq repositories is typically confined to precomputed gene expression tables [1, 2]. As it is restricted to annotated genes or transcripts, this approach overlooks a large part of transcriptional diversity, which includes mutated, abnormally spliced, intergenic, intronic, repetitive, or fusion RNAs [3]. Projects such as Recount offer a way to query independent exons or splice junctions in very large (SRA-scale) datasets [4]; however, this still relies on sequence alignments and does not allow to quantify an arbitrary RNA directly. Considering the huge diversity of RNA forms, searching RNA-seq repositories using current tools is like looking under the proverbial lamppost. New methods are required to explore the hidden diversity within RNA-seq data.

Reference-free queries in large sequence sets are possible thanks to several k-mer-based data structures that can index large sequence datasets in a fraction of the disk space used for raw sequences (see [5] for review). However, most k-mer data structures are limited to qualitative queries (presence or absence of a given sequence), which is not satisfying for RNA expression analysis. Three recent tools enable quantitative queries in large sequence sets. Needle [6] implements multiple interleaved Bloom filters and sketches of minimisers, which enable storing counts in a semi-quantitative way. Metagraph [7] uses an optimized De Bruijn Graph structure, enabling to store either presence-absence or count information. While Metagraph proposes ready-made indexes for diverse collections of genomes and metagenomes, the public server does not return count information and is limited to one query sequence at a time. Our indexing tool Reindeer [8] is optimized for processing several thousands of samples and associate k-mers to approximate but accurate counts in each sample.

Our collaborative group has been working under an umbrella project named Transipedia, aimed at facilitating reference-free transcriptome analysis through improved RNA-seq indexes. Here, we use an improved version of Reindeer deployed on a web server to demonstrate the capacity of reference-free RNA-seq indexes to detect and quantify arbitrary RNA variations of biological significance in cancer RNA-seq data. First, we re-evaluate the computational time and memory footprint of Reindeer in this practical setting. We then show that transcript quantification with Reindeer can achieve a high accuracy by masking non-specific sequences in queries. Building upon this, we introduce the first public reference-free index of the CCLE RNA-seq database. The rich biological data in CCLE (1019 cell lines from 40 tumor types) allows us to illustrate Reindeer’s ability to accurately detect and quantify a large diversity of non-reference RNA sequences, including RNA mutations, fusions, transposable elements, and splice variants. The reference-free CCLE RNA atlas is available for online queries along with other datasets at https://transipedia.org.

## Results

### Indexes for arbitrary RNA sequence query and quantification

Our objective is to provide a computational framework enabling quantification of arbitrary RNA sequences in large RNA-seq datasets. This framework must satisfy several criteria: (i) the capability to index any RNA-seq dataset while preserving all information at single-base resolution and (ii) the ability to query the index in real-time for quantifying the occurrence of input sequences in each sample within the index. Indexes should be available for query either through a web interface or on a local computer. We describe below the realization of such a framework using Reindeer.

### Building and querying indexes

**Fig. 1:**
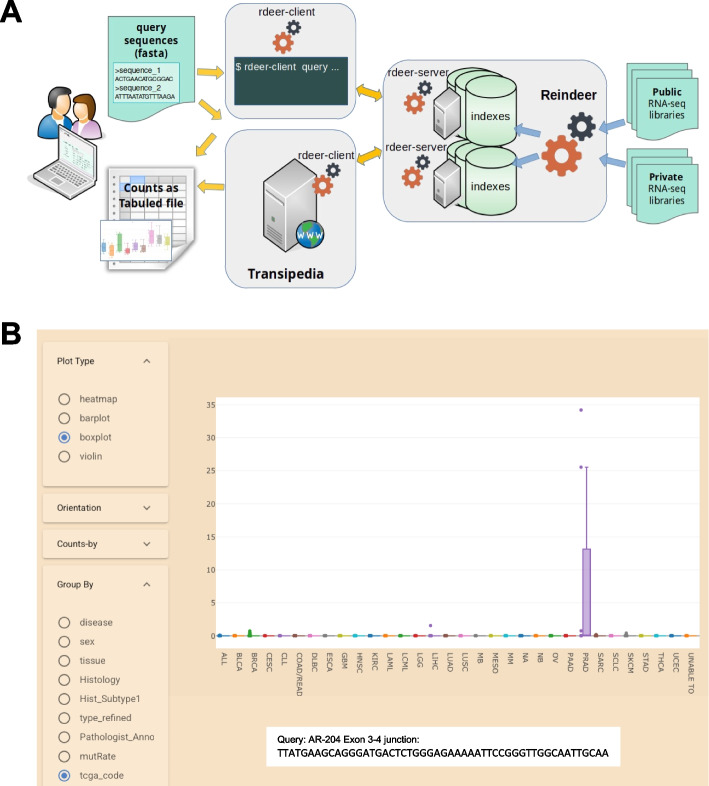
**A** Reindeer index build and query workflow implemented on the Transipedia web server. Reindeer pre-built indexes can be easily queried by nonexperts using the Transipedia web interface. For large queries and/or pipelines, locally installed indexes can be queried from the command line using rdeer-client (rdeer). The output is a tabulated count file. **B** Graphical output of a query corresponding to a prostate cancer-specific sequence (AR-V7). Each dot corresponds to a cell line, with Reindeer counts on the *Y* axis. The query sequence was a 51-nt fragment spanning exon 3-4 junction, specific to androgen receptor variant AR-V7 (Gencode transcript AR-204). Examples of web queries are provided on the repository: https://github.com/Transipedia/Reindeer-use-cases

The implementation of a Reindeer index server is presented in Fig. 1A. Indexes were created with a k-mer size of 31, using the on-disk option that allows queries to be performed while only storing the primary k-mer hash in memory. Currently available online indexes cover 151 billion reads in 1851 samples. Indexes have relatively small memory footprints and file sizes 15 to 40 times smaller than the original compressed fastq files (Table 1). For instance, hardware requirements for querying the 1019-sample CCLE index are only 22.3 Gb RAM and 236 Gb disk. A socket mode enables the index to reside in memory once loaded and allows for real-time queries. Users submit queries through the web interface and receive results in the form of count tables or graphics (Fig. 1B). Query times are fast enough to handle multiple interactive queries for short sequences or full-length mRNAs (100,000 31-mers in 16s, 100 mRNAs in 14.5s) (Table 2).

**Table 1:** Reindeer index properties for various datasets (on-disk indexes)

Dataset	#Samples	Fastq.gz size (Gb)	Index size (Gb)	RAM (Gb)	Load time (h:m:s)
GSE62852-AML	40	252	16	10.8	00:02:17
GTEx (part)	1119	6100	312	42.2	00:08:58
SEQC/MAQC	16	51	2.4	3.1	00:00:33
CCLE	1019	8900	236	22.3	00:05:57

**Table 2:** Query times on the CCLE index (on-disk index)

Query type	# Queries	Query time (s)
31-mers	1000	1.0
	10000	2.0
	100000	16.0
	500000	89.0
	1000000	179.0
Full-length mRNAs (mean size: 1.9 kb)	1	0.6
	100	14.5
	1000	132.8

### Accuracy of RNA expression measure

**Fig. 2:**
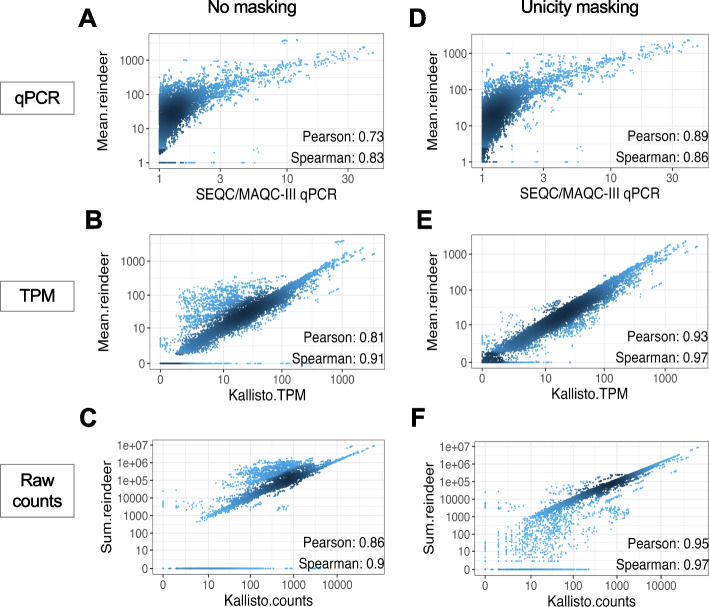
Correlations between Reindeer counts and established count methods. 1000 genes were quantified in 16 reference SEQC/MAQC-III samples. **A**, **D** Reindeer (mean counts) vs. qPCR. **B**, **E** Reindeer (mean counts) vs. Kallisto TPM. **C**, **F** Reindeer (sum counts) vs Kallisto raw counts. Unicity masking: counts obtained after removal of non-unique k-mers

In order to assess Reindeer’s capacity to accurately quantify RNA expression from RNA-seq samples, we compared it to standard quantification approaches. Reindeer queries can be made using full-length sequences (e.g., complete mRNAs) or fragments of size not smaller than *k* as input. Reindeer returns counts for all consecutive k-mers in the query (Additional file 1: Fig. S1, Additional file 3: Supplementary Methods). Counts can be interpreted in different ways depending on whether users expect raw counts or counts normalized by query sequence length. To determine the optimal counting scheme, we used the SEQC/MAPQC dataset in which the abundance of 1000 transcripts was evaluated in 16 samples both by qPCR and Illumina RNA-seq [9]. Means of k-mer counts best correlated with qPCR abundance and transcript-per-million (TPM) measured from RNA-seq reads by Kallisto [10] (Fig. 2A, B, Additional file 1: Fig. S2), while sums of k-mer counts best correlated with raw RNA-seq counts (Fig. 2C, Additional file 1: Fig. S2). Correlation coefficients (CC) with Kallisto counts were around 0.8, in line with previous reports [6]. We found that quantification accuracy could be substantially improved by masking query k-mers with multiple instances in the human genome (“Methods”). This procedure led to > 0.9 Pearson correlations with both qPCR and RNA-seq derived abundances, reaching a Pearson CC of 0.95 with Kallisto raw counts (Fig. 2D–F, Additional file 1: Fig. S2). This demonstrates that simple quantitative queries in a k-mer index can achieve accuracies approaching that of a state-of-the-art RNA-seq quantification method. Note that while TPM-like counts are identical in absolute value across methods, raw counts require a linear correction due to the conversion of fragment to k-mers (discussed in Additional file 3: Supplementary Methods).

### Finding mutations in RNA

Given an index of 1019 cancer cell lines enabling fast and accurate quantification of arbitrary RNAs, we set out to use this system to retrieve different types of RNA variations not commonly accessible in transcriptome databases. First, we designed queries for mutations and indels. We selected mutations/indels in common cancer genes from the Depmap database [11] and designed 61-nucleotide sequences around each variation as explained in the “Methods” section (Additional file 1: Fig. S3, Additional file 2: Table S1). We refer to these sequences as “probes.” With *k *= 31, a 61-nucleotide ($2k-1$) size ensures that any k-mer in the probe covers the variation.

To limit false positive calls, we applied a masking step that discarded parts of probes with multiple hits in the genome or harboring low complexity sequences (“Methods”). While this only eliminated 1.7% of probes (Table 3), it reduced false positive hits by 93.9% (Additional file 2: Table S2). Several query modes were then tested whereby at least 1, 3, 5, or 10 k-mers in each probe had to be non-zero for the call to be made ($min\_hits$ = 1 to 10) (Table 3). Recall was satisfying in all cases (0.875 to 0.945), while precision ranged from 0.269 ($min\_hits$ = 1) to 0.893 ($min\_hits$ = 10). Thus, there is a significant benefit in requiring several k-mer hits around an event to make a call. Hereafter, $min\_hits$ is set to 3 unless specified otherwise.

**Table 3:** Accuracy measures of Reindeer mutation and fusion calls

		#total probes	#probes after selection^a^		*min_hits*^c^*=1*	*min_hits=3*	*min_hits=5*	*min_hits=10*
Mutations	All Depmap mutations^b^ (50 cancer genes)	3685	3621	^d^True +	4346	4255	4205	4026
				False +	11810	911	589	**484**
				False -	**255**	346	396	575
				Precision	0.269	0.824	0.877	**0.893**
				Recall	**0.945**	0.925	0.914	0.875
	Cosmic Hotspot mutations	960	951	True +	1665	1631	1611	1558
				False +	6823	184	114	**90**
				False -	**51**	85	105	158
				Precision	0.196	0.899	0.934	**0.945**
				Recall	**0.970**	0.950	0.939	0.908
Fusions	All Depmap fusions (junction at exon edges)	8972	8860	True +	9410	9277	9201	9018
				False +	25732	10048	6558	**2378**
				False -	**170**	303	379	562
				Precision	0.268	0.480	0.584	**0.791**
				Recall	**0.982**	0.968	0.960	0.941
	Cosmic fusions	60	59	True +	99	98	98	96
				False +	22	3	3	**2**
				False -	**1**	2	2	4
				Precision	0.818	0.970	0.970	**0.980**
				Recall	**0.990**	0.980	0.980	0.960

^a^Min count cutoff and low complexity masking

^b^Restricted to RNA-seq-derived mutations

^c^*min_hits:* minimum number of positive k-mers in query

^d^All positive and negative counts are given for pairs $\{$probe, sample$\}$

**Fig. 3:**
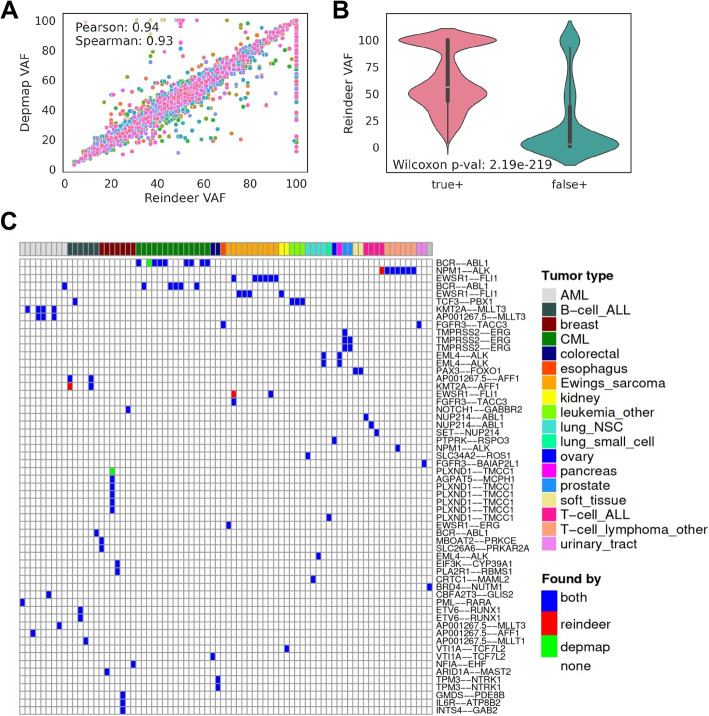
Comparison of Depmap and Reindeer calls for mutations and fusion transcripts. **A**, **B** Reindeer variant allele frequencies (VAF) measured as the count ratio: mutated / (mutated + wild-type) * 100, for all Depmap mutations in cancer genes. **A** Correlation of Depmap VAF (based on RNA-seq alignment) and Reindeer VAF. Each dot shows a mutation in one sample, colored according to gene (50 genes). **B** Comparison of Reindeer VAF for true positive (*n* = 4255) and false positive (*n* = 911) calls in Depmap. **C** Detection of DepMap Cosmic fusion events in CCLE cancer cell lines. Cosmic fusions were retrieved using a 51-nt probe centered on the fusion junction. Top: cell lines are colored by tumor type. Blue: events from DepMap found by Reindeer (true positive); red: events found in an extra sample with Reindeer compared to DepMap; green: events not found with Reindeer. Lines with identical fusion names correspond to different exon-exon junctions of the same genes

Restricting Reindeer queries to recurrent (“hotspot”) cancer-related mutations from Cosmic [12] substantially improved precision and recall ($\ge$ 0.9, Table 3). We hypothesized that the remaining false positive (FP) calls may be true mutations filtered out by Depmap due to a more stringent count threshold. To assess this, we computed the variant allele frequencies (VAF) of mutations using counts obtained with wildtype and mutant probes. VAF computed by Reindeer was in general highly correlated to that inferred from conventional RNA-seq alignment (Fig. 3A) and FP calls had significantly lower VAF (Additional file 2: Table S3, Fig. 3B), supporting these may be in part censored by Depmap. Further testing of 12 RNA-seq files (corresponding to 78 FP pairs) using a sensitive variant caller [13] or by direct parsing of the fastq files confirmed 76 of the 78 (97%) of the putative FPs as likely true positives (Additional file 2: Table S4). Finally, we analyzed samples with available DNA sequencing data: out of 44 FP calls in these samples, 31 (70%) turned out positives at the DNA level (Additional file 2: Table S5). In summary, we estimate that the majority of the putative FP mutations at $min\_hits$=3 are actually true mutations.

### Finding fusion transcripts

We next tested Reindeer’s capacity to retrieve gene fusion events. DepMap provides genomic coordinates of fusion junctions identified after alignment of RNA-seq reads by STAR-fusion [14]. We selected fusion events with a breakpoint at exon edges, which are considered more reliable [15], and designed 51-mer sequences centered on the fusion junction (“Methods,” Additional file 1: Fig. S3B & Additional file 2: Table S6). Probes shorter than $(2k-1)$ are desirable when querying fusion and splice junctions, since k-mers overlapping the junction at their tip might accidentally match other partner exons. Masking of k-mers present in the reference genome or transcriptome and of low complexity k-mers (see the “Methods” section) yield a total of 8860 fusion probes (Additional file 2: Table S2).

Fusion events were quantified requesting at least 1, 3, 5 or 10 non-zero count k-mers, as done for mutations (Table 3). Recall was high in all cases (0.94 to 0.98), but precision was relatively low (0.27 to 0.79) due to a high number of FPs. Restricting evaluation to Cosmic fusions (100 fusion events) largely reduced the FP rate, improving both precision and recall to above 0.97 for $min\_hits\ge 3$ (Table 3, Fig. 3C). This suggests the initial query list from Depmap contained fusions yielding multiple erroneous hits. The only two missed fusion events had SNPs in close proximity (7 and 4 nucleotides) to the junction, such that the minimum number of matching k-mers was not reached (Additional file 1: Fig. S4). Of the three apparent false positives remaining (Fig. 3C, red), two were annotated in the LigeA fusion database [16] in the correct cell line, supporting their reality. Finally, fusion transcript expression quantified by Reindeer was highly correlated to that given by Depmap (Pearson CC = 0.92, Additional file 1: Fig. S5).

### Finding expressed transposable elements

**Fig. 4:**
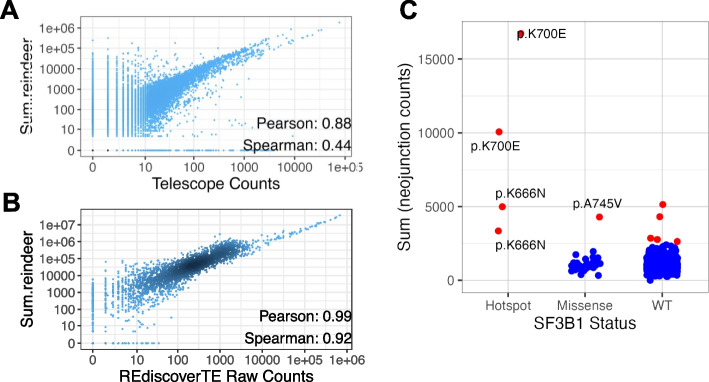
Quantification of transposable elements and novel splice junctions. **A** Correlation of quantification of 1000 ERVs by Reindeer and Telescope, in 56 colon cell lines from CCLE. **B** Correlation of quantification of 50 ERV families by Reindeer and RediscoverTE, in 56 colon cell lines from CCLE. **C** Quantification of SF3B1-induced neojunctions in cell lines. Each dot represents the sum of counts of 849 SF3B1-induced neojunctions in one CCLE cell line. Cell lines harboring hotspot (likely oncogenic) SF3B1 mutations, other missense SF3B1 mutations and wild-type SF3B1 are distinguished. Cell lines with outlier neojunction expression (> 2 SD above mean) are shown in red

Transposable elements in the human genome are mostly silent but can be re-expressed in tumor cells upon lifting of epigenetic repression. Measuring their expression is complex because exact repeats impede the attribution of RNA-seq reads to specific loci. We compared the quantification of human endogenous retroviruses (ERV, a major class of transposable elements) by Reindeer and by two software relying on different mapping strategies. Telescope [17] estimates transposable element expression at locus-level through genome mapping, allowing for up to 100 mapping positions and reassigning ambiguous reads to specific loci using an expectation maximization algorithm. While Reindeer does not use expectation maximization, locus-level ERV quantification after masking of non-unique sequences was reasonably similar to that of Telescope (Pearson CC:0.88, Fig. 4A, Additional file 1: Fig. S6), while requiring only a fraction of the time (4–5 h by sample with Telescope vs. seconds for Reindeer). REdiscoverTE [18] estimates transposable element expression at the family level based on Salmon [19], a fast quantifier using pseudo-mapping. REdiscoverTE and Reindeer ERV quantifications were highly correlated, both for raw counts and for CPM normalized counts (Pearson CC = 0.99 and 0.96 respectively) (Fig. 4B, Additional file 1: Fig. S7). Although a significant fraction of k-mers in ERV elements were masked as non-unique (“Methods”), all tested elements had sufficient specific k-mers to remain quantifiable, even at the locus level. Finally, as observed for mRNA quantification, Reindeer’s raw counts required a linear correction to match raw counts from the specialized tools.

### Finding aberrant splicing junctions

Aberrant splice junctions caused by mutations in RNA processing genes are generally absent from reference transcriptomes. Their detection usually requires downloading and reanalyzing RNA-seq files. Using Reindeer, one may directly interrogate an RNA-seq index for such unreferenced variants. We illustrate this with splicing alterations in uveal melanoma. Mutation of the SF3B1 splice factor in uveal melanoma induces aberrant splicing of hundreds of genes [20]. We retrieved aberrant splice junctions observed in SF3B1-mutated patients and created 51 nucleotides probes for 849 so called neojunctions (see the “Methods” sections). These sequences were then quantified in CCLE (Fig. 4C). All cell lines harboring known oncogenic SF3B1 mutations presented significantly elevated neojunction expression, consistent with genome-wide SF3B1-induced alterations. Another SF3B1 mutation with elevated neojunctions was A745V in NCIH358_LUNG, suggesting this mutation may also disrupt splicing, although this is not documented in the current literature. Moreover, five cell lines with no SF3B1 mutation behaved like SF3B1 hotspot mutants, suggesting alterations in the same splicing pathway in these cells. This included two lung and two endometrial tumors, which are tumors where SF3B1 and related SUGP1 mutations are documented [21, 22]. Interestingly, two of these cell lines had impairing mutations in SUGP1 (Additional file 2: Table S7). This illustrates how a Reindeer index can be utilized to evaluate a complex transcriptome signature composed of aberrant transcripts and identify cells altered in similar pathways.

### Transferring probes across datasets

Finally, we assessed how reliably probes designed from one dataset could be used for querying other datasets. We used the above Depmap-derived probes to query two cancer sample datasets with known ground truth. Mutations were queried in a lung adenocarcinoma dataset (*N *= 77) [23] and fusions in a leukemia dataset (*N *= 148) [24–26]. Overall, we found that mutations and fusions in the independent datasets were reliably identified with $> 90\%$ recall and precision (Additional file 1: Fig. S8, Additional file 2: Table S8).

## Discussion

We describe here the first practical implementation of a web server for reference-free, quantitative queries in an RNA-seq dataset of over 1000 samples. The service runs on a standard computer using less than 25 Gb memory and 250 Gb SSD storage. It was tested with a variety of input queries including full-length mRNAs and RNA elements that are not usually represented in curated RNA-seq databases, such as transposable elements, fusions, neo-splice junctions, and mutated RNAs. When using the system to retrieve known mutation and fusions events, precision and recall were above 0.9 for oncogenic events. Furthermore, a large fraction of inferred false positives were shown to be likely true events filtered out in the reference database.

Reindeer count accuracy was high in spite of the conversion of read counts into aggregated k-mer counts at indexing and their subsequent conversion to query-level counts when processing query results. Count correlation with state-of-the-art quantification methods were always above 0.8 (Pearson CC) for full-length mRNAs, fusion transcripts, and transposable elements and above 0.9 after masking non-specific k-mers from queries.

A lesson learnt during this study was the importance of “query engineering,” i.e., proper probe design, masking, and post-processing. Query design involves selecting the right “probes” to ensure returned hits do not include unspecific sequences. With our default k-mer size of 31 nucleotides, optimal probes were 61-nt fragments around mutations or 51-nt fragments around splice or fusion junctions. Query masking involved removal of non-specific (non-unique and low complexity) k-mers from queries. This provided important gains in count accuracies for all types of queries. Furthermore, this considerably reduced the number of false positives when querying local events such as mutations. The query design and masking methods introduced herein could serve as guidelines to users of k-mer-based indexes in general.

Post-processing of query results first involves deciding how many k-mers in a query must be matched to accept a hit. This step is only important for local event detection (mutations, fusions, splice junctions), in order to accomodate possible SNPs around events. We identified the optimal setting whereby flanking SNPs minimally interfered with mutation calling while retaining a high specificity. The second post-processing step is the conversion of Reinder k-mer counts into TPM-like or raw-count-like values. Averaging k-mer counts provided count estimates that were remarkably similar to TPM, while summed counts were highly correlated to raw counts, albeit with a conversion factor.

Some limitations of the current Reindeer framework must be acknowledged. (i) Reindeer index building is a separate action that is computer intensive and involves resolving a few technical challenges, such as read quality control and trimming. (ii) Real-time queries are available to web users thanks to preloaded indexes. Tools for pre-loading indexes are provided in the “Methods” section. However, local instances will have to load indexes into memory first, which may take several minutes before queries are processed. (iii) Query design may require running an independent tool such as the *Kmerator Suite* [27] prior to submitting queries. This may be further integrated into the server after enough user experience is gathered.

## Conclusion

Reference-free indexes provide a direct access to unprocessed RNA-seq data, enabling biologists to ask questions that would otherwise require resource-intensive pipelines. Beyond obvious applications such as verifying the tissue or tumor specificity of novel biomarkers, Reindeer’s quantitative indexes allow to carry out sophisticated experiments by simultaneously querying oncogenic alleles, RNA isoforms, repeats, etc., and process the resulting count table to uncover novel functional interactions. We hope to expand the Transipedia server to include an increasing number of public datasets to facilitate this type of experiment.

## Methods

### Updates to Reindeer

Since its initial publication [8], Reindeer has been enhanced with a socket mode to facilitate remote server queries. This improvement enables the efficient management of indexes from various collections and ensures rapid query responses. Reindeer utilizes an efficient k-mer hashing structure to map k-mers to their respective counts in each sample, alongside a matrix that represents the abundance of indexed objects across samples. Through extensive testing, we observed that the primary bottleneck in many use cases was the loading of the index into RAM, while the actual querying process is quickly expedited thanks to the hashing structure. As a result, Reindeer’s default algorithm was transitioned from relying predominantly on in-RAM queries to disk-based queries. This shift involves the ability to serialize the count matrix of Reindeer, its most expensive part, onto the disk in a compressed format. Conversely, the hashmap has a reduced footprint thanks to an efficient hash function and co-encoded keys. Consequently, we updated Reindeer to only load the hashmap into RAM in the initial phase, and read lines of the count matrix only when necessary, markedly reducing the total time required for conducting intensive queries, especially when running on SSD. 

### Building and using Reindeer indexes

Building codes for the web and local server environment are described in https://github.com/Transipedia/publication-ccle. For RNA-seq data sources, see Data Availability and Table S9. CCLE RNA-seq raw fastq files were retrieved from Gene Expression Ominibus dataset GSE36139. Fastq files were first checked for sequence quality using FastQC (version 0.11.9), MultiQC (version 1.9) and KmerExplor [27] for contaminations and library information. Cutadapt (version 1.18) was used for low-quality trimming (-q 10,10), excluding sequences shorter than 31 nt after trimming (-m 31). Adapter sequence removal was deemed unnecessary in the studied datasets. Fastq files were then processed by bcalm v2.3.0 (https://github.com/GATB/bcalm). For the CCLE dataset, k-mers with counts $<4$ were excluded (option -abundance-min 4). Bcalm files were then used as input to Reindeer v1.02 (https://github.com/kamimrcht/REINDEER). Indexes for the web server were built using the on-disk option. For querying, indexes were copied to an SSD drive (applies to the web server too). All query times were obtained using the rdeer-client software running on a local index and include count aggregations for multi-probe queries.

### Gene expression quantification benchmark

The SEQC/MAQC-III dataset [9] provides both RNA-seq and qRT-PCR values for 1000 genes across 16 reference samples. We used the 16 Illumina files and the pre-processed Taqman-raw.txt file from Chisanga et al. [28], retrieved from https://github.com/ShiLab-Bioinformatics/GeneAnnotation. RNA-seq data was processed as above. Gene expression was quantified with Kallisto (version 0.46.1) using the v108 Ensembl transcriptome (cdna+ncrna), followed by tximport [29] for computing gene-level raw counts and TPM values. The Reindeer index was generated from trimmed fastq files using cutadapt -q 10,10 -m 31. Reindeer gene expression estimates were obtained using Ensembl v108 canonical transcripts as input, subject to the following processing steps.

### Query preparation

Queries were pre-processed to remove non-specific and low complexity k-mers. When “masking” is specified, non-specific parts of query sequences were deleted using the Kmerator software (https://github.com/Transipedia/kmerator) [27]. Kmerator takes as input a genome index and a fasta file of query sequences or a list of gene names. By default any k-mer in the query that is present more than once in the genome is deleted. The optional parameter –max-on-transcriptome X requires that k-mers be present at most *X* times in the transcriptome annotation file (Ensembl v108 was used).

Low complexity masking discards k-mers meeting any of the following conditions:

- Containing a $\ge 6-$nt homopolymer, or
- 3-mer complexity defined as ((number of distinct 3-mers in k-mer)/(total number of 3-mers in k-mer)) below 0.55 in k-mer. This cutoff was determined from the analysis of complexity distribution in four independent datasets (Additional file 1: Fig. S9).

### Processing of query results

Reindeer queries return a series of triplets $b_i-e_i:q_i$, each corresponding to a monotig (Additional file 1: Fig. S1 and Additional file 3: Supplementary Methods) matched by the query sequence. A $*$ symbol for $q_i$ means that the monotig does not have enough k-mers (with non-zero counts) for reporting a reliable result. This minimum k-mer presence criteria is provided as a percentage in the $-P$ parameter. The default $-P$ value (40%) was used unless otherwise specified. Query abundance (Fig. 2A–C and Additional file 1: Fig. S2, left) was computed as the mean, median, maximum, and sum values of monotig counts. Mean, median, and sum were weighted by the number of k-mers in each monotig. The maximum value was calculated in the trivial way as it is not affected by k-mer multiplicities. For masked queries (Fig. 2D–F, Fig. 4A, B, Additional file 1: Fig. S2 right, Additional file 1: Fig. S6, Additional file 1: Fig. S7 and Additional file 1: Fig. S10A), substrings were queried separately by Reindeer and the resulting counts were merged per original query (this option is available on the web server; however, it is only possible with *mean* abundance counting).

### RNA mutations

Fifty highly mutated cancer genes were extracted from CCLE [30], TCGA [31], and hematological malignancies [32] (Additional file 2: Table S10). RNA-seq derived mutations within these genes were retrieved from the DepMap Public 22Q2 MAF mutation file (file CCLE_mutations.tsv, field RNAseq_AC) and converted into VCF format. This represented 3685 mutations, herein referred to as “all Depmap.” A subset of 960 probable cancer drivers was further selected based on field CosmicHotSpot in the mutation file. The probe selection process for mutations is described in Additional file 1: Fig. S3A. For each mutation, a 61 nt-long probe centered on the mutation and its wildtype 61 nt-long counterpart were produced with *vcf2seq* https://github.com/Bio2M/vcf2seq. Mutant probes were masked using Kmerator with the –chimera option that deletes any k-mer present in the reference genome or transcriptome, and wild-type probes were masked with the –max-on-transcriptome 100 option that only deletes non-unique k-mers on the genome and > 100 occurrences on the transcriptome. Probes were also masked for low complexity elements as described above. At the end of the masking process, about 2% and 1% of probes were deleted from the “all Depmap” and hotspot probe sets, respectively (Table 3). To assess putative false positive calls, we selected 12 samples corresponding to 78 FPs and performed variant calling using the Crac alignment software [13] combined to CracTools (http://crac.gforge.inria.fr/). We also directly parsed fastq files using CountTags (https://github.com/Transipedia/countTags), extracted corresponding reads, and aligned them to the hg38 reference genome using Blat [33]. Additional file 2: Table S4 reports putative false positives evaluated as true positives through either method.

### Fusions

Fusions were retrieved from the DepMap Public 22Q2 fusion table (field CCLE_fusions.csv). We set the minimum read count supporting a fusion to 4 (same as used in the Reindeer index) which retained 14946 fusions. A bed file was generated for the left and right sides of junctions and 51 nt-long probes centered on the junction were produced using bedtools getfasta[34]. We then selected fusions with junctions at exon edges by intersecting fusion coordinates with Gencode V42 exon coordinates (8972 fusions). K-mers were masked using kmerator –chimera and low-complexity filter as above. The complete procedure is shown in Additional file 1: Fig. S3B. A total of 8860 fusion queries were eventually retained. A subset of 60 known oncogenic fusions was selected based on the “Cosmic” label in column annots of the DepMap table. Selected fusions were further verified on the Ligea dataportal (http://hpc-bioinformatics.cineca.it/fusion/) which provides fusions predicted in CCLE RNA-seq data by four detection algorithms and enables retrieval of the corresponding read sequences.

### Transposable element expression

Transposable element quantification was performed in 56 CCLE samples from colon cell lines. For comparison with Telescope [17] (V.1.0.3), we selected 1000 ERV loci (4034 sequences) from the authors’ supplemental data. We then generated query sequences based on genomic coordinates (Hg38), and masked non-unique sequences using kmerator with option –max-on-transcriptome 100. Unicity masking deleted 17% of k-mers in ERV probes in average. Nonetheless, every locus retained at least one probe with enough specific k-mer to be measurable. Telescope runtime was estimated based on a run with 16 threads and 48Gb RAM. For tests against REdiscoverTE [18], we retrieved genomic locations for 58 ERV families from the adapted REdiscoverTE data available at https://github.com/ucsffrancislab/REdiscoverTE/. This represented 40,734 loci, which were converted to sequences using bedtools, and masked for non-unique k-mers as above, resulting in 305,331 probes. Counts were aggregated at the family level.

### Neo-splicing events

The coordinates of 1258 abnormal splice junctions associated to SF3B1 mutations were retrieved from Table 2 of [20], converted to bed format and lifted to Hg38 using overlift (UCSC tools). As in the fusion procedure, we generated a 51-nt long sequence centered on the splicing junction and masked any genome or transcriptome k-mer (kmerator –max-on-transcriptome 0) and low complexity k-mers, retaining 849 probes. Probes were quantified in CCLE using the mean method.

### Querying across datasets

For independent validation of Depmap-derived probes into independent datasets, we built Reindeer indices for 77 lung adenocarcinoma samples (together with 77 matched normal samples) [23] and 148 leukemia samples [24–26] and queried them with minimum counts set to 3. Accessions are listed in the Data Availability section. Ground truth mutation and fusion calls in each sample were retrieved from the original publications. Depmap probes were available for 32 mutations (in total 50 ground truth mutation-sample pairs) in the lung cancer dataset and 7 fusions (in total 63 ground truth fusion-sample pairs) in the leukemia dataset.

## Supplementary information

Additional file 1: Supplementary figures. Fig. S1. Principle of Reindeer counting. Fig. S2. Correlation between Reindeer counts and established count methods. Fig. S3. Design of mutation/fusion probes and quantification. Fig. S4. Alignment of false negative Cosmic fusion probes. Fig. S5. Correlation between Junction read count from DepMap and Reindeer raw counts. Fig. S6. Correlation between Reindeer and Telescope counts. Fig. S7. Correlation between Reindeer and REdiscoverTE counts. Fig. S8. Matches of Depmap-derived probes in independent datasets. Fig. S9. Distribution of 31-mer complexity. Fig. S10. Analysis of correlations between Reindeer sum counts and raw counts from other tools.

Additional file 2: Supplementary tables. Table S1. 61-nt mutation probes. Table S2. Effect of unicity and low complexity masking on mutation and fusion calls. Table S3. List of the 911 False positive mutations calls. Table S4. Subset of 78 putative false positive mutations reanalysed with CRAC or countTags. Table S5. Subset of 44 putative FP mutations analyzed at the DNA level by WES data. Table S6. 51-nt fusion probes. Table S7. CCLE cell lines with high countsof SF3B1-related neojunctions. Table S8. Results of cross-cohort queries. Table S9. RNA-seq datasets used in study. Table S10. List of cancer genes used for mutation detection.

Additional file 3: Supplementary Methods.

Additional file 4: Review History.

## Data Availability

Publicly available data were obtained from the Gene Expression Omnibus accessions GSE62852 [35], GSE47792 [36], GSE36139 [37], and GSE40419 [38] and ENA accessions PRJNA265845, PRJNA523380, PRJNA265845, and PRJEB3132 respectively. Leucegene data were obtained from GSE49642 [39], GSE52656 [40], and GSE62190 [41] and ENA accessions PRJNA214592, PRJNA229548, and PRJNA263397. The restricted access GTEx dataset was obtained from dbGAP (phs000424.v8.p2) with authorization to T.C. [42]

## Code availability

Code is available in Zenodo https://zenodo.org/records/13819530 [43] and a GitHub repository https://github.com/Transipedia/publication-ccle [44].
