# Supplementary material for: Transipedia.org: k-mer-based exploration of large RNA sequencing datasets and application to cancer data
Source: Genome Biol. 2024 Oct 10;25:266. doi: 10.1186/s13059-024-03413-5 (PMC11468207; doi:10.1186/s13059-024-03413-5)
Supplement: Supplementary file 1 — Additional file 1: Supplementary figures. Fig. S1. Principle of Reindeer counting. Fig. S2. Correlation between Reindeer counts and established count methods. Fig. S3. Design of mutation/fusion probes and quantification. Fig. S4. Alignment of false negative Cosmic fusion probes. Fig. S5. Correlation between Junction read count from DepMap and Reindeer raw counts. Fig. S6. Correlation between Reindeer and Telescope counts. Fig. S7. Correlation between Reindeer and REdiscoverTE counts. Fig. S8. Matches of Depmap-derived probes in independent datasets. Fig. S9. Distribution of 31-mer complexity. Fig. S10. Analysis of correlations between Reindeer sum counts and raw counts from other tools. [file 13059_2024_3413_MOESM1_ESM.pdf]

Supplementary Figures for:

Transipedia: k-mer based exploration of large RNA sequencing datasets and application to cancer data

Bessière *et al*. 2024

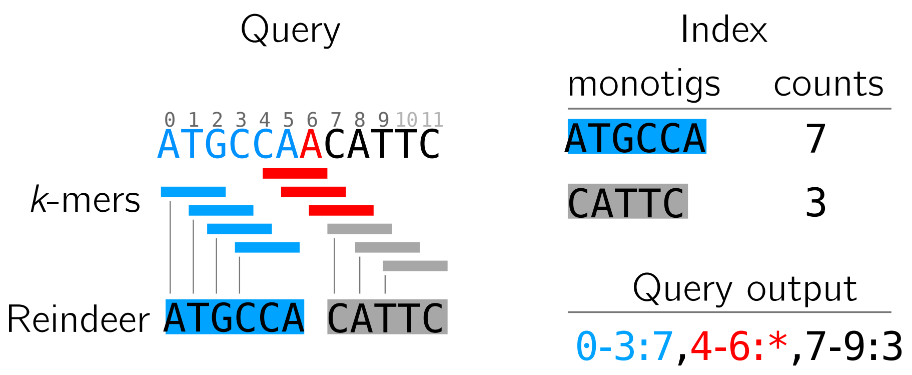

Fig S1: Principle of Reindeer counting. Reindeer natively reports counts for one sample, based on k-mers in the query matched to k-mers in a given monotig index. Monotigs are Reindeer's subunits that aggregate consecutive k-mers with same counts, they allow to associate a single count to a series of k-mers. In the example, k-mer size is 3 and the index has two monotigs.

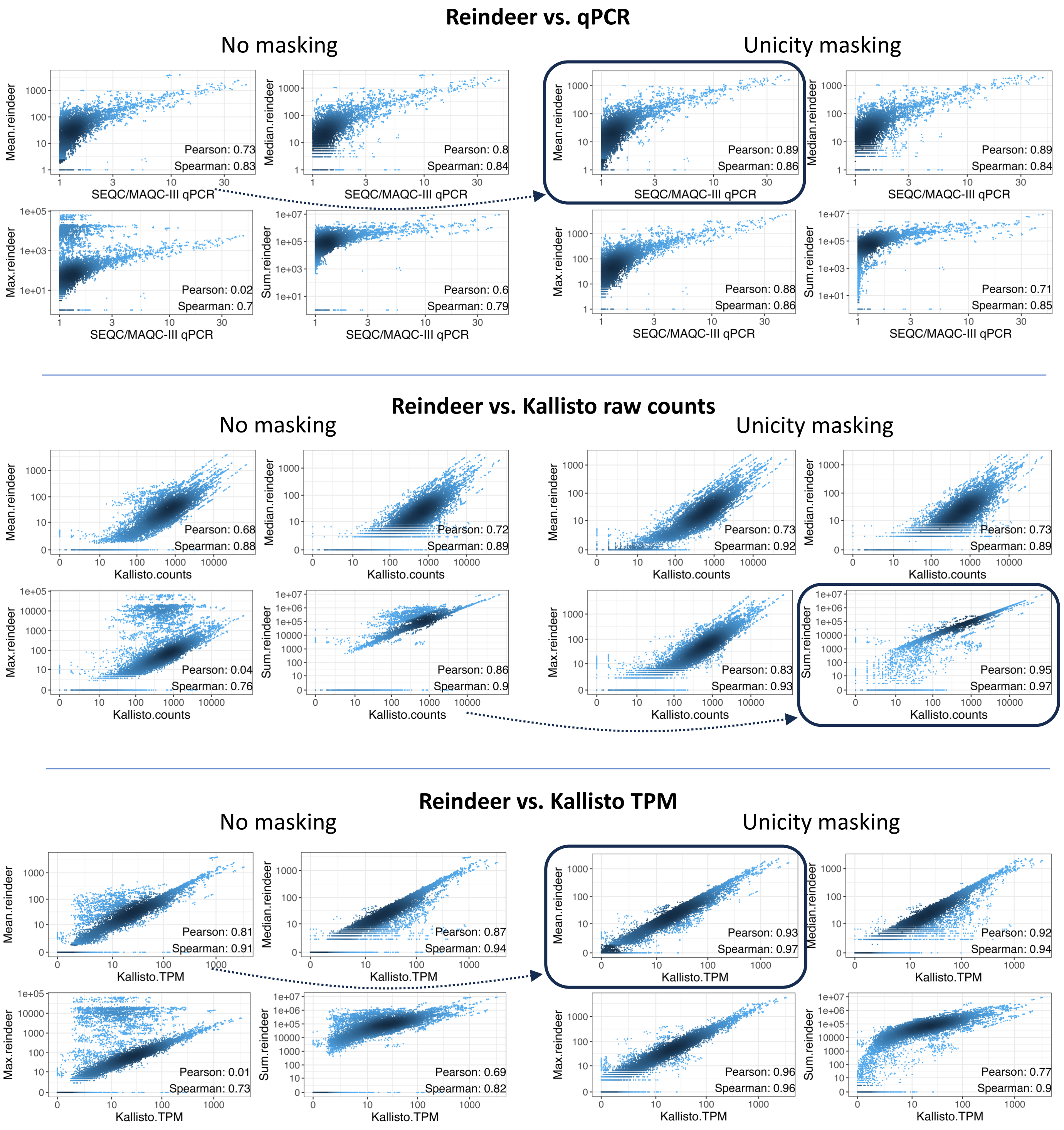

Fig S2: Correlation between Reindeer counts and established count methods, for 1000 genes quantified in 16 reference SEQC/MAQC-III samples. For each comparison, Reindeer counts are computed using one of 4 methods: mean, max, median or sum of monotig counts. On the right side ("masking") query sequences were first masked through removal of all non-unique k-mers.

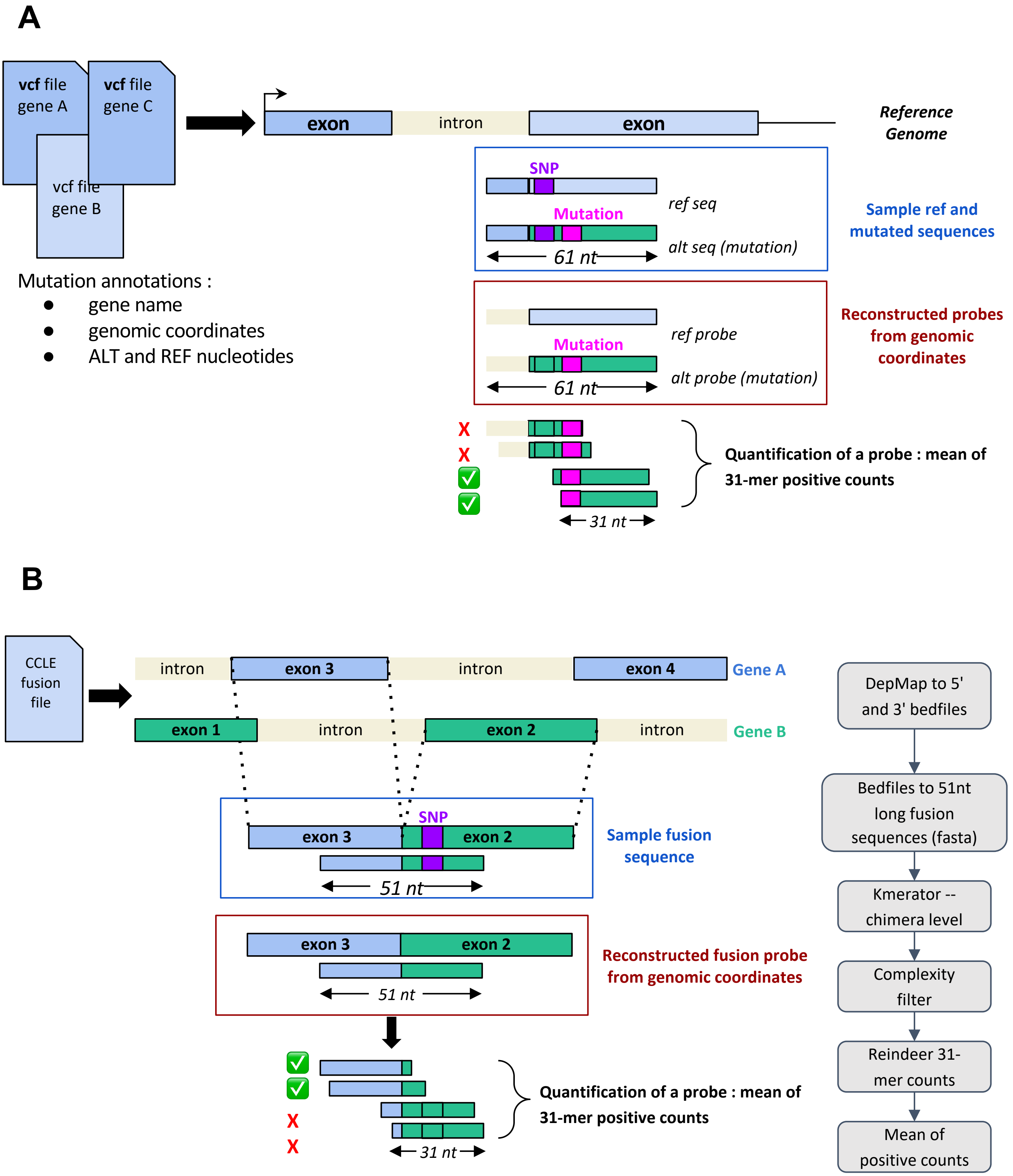

Fig S3: design of mutation/fusion probes reconstructed from genomic coordinates and quantification. **A:** design of 61nt probes for mutation queries. These probes are decomposed into 31-mers for quantification. **B:** design of 51nt probes for fusion queries. These probes are decomposed into 31-mers for quantification. The workflow on the right includes filtering for unicity (Kmerator with --chimera level) and complexity.

```
chr22:23289621:+_chr9:130854064:+_BCR--ABL1_NALM1_Probe     -----CATTCCGCTGACCATCAATAAGGAAGAAGCCCTTCAGCGGCCAGTAGCATC----
chr22:23289621:+_chr9:130854064:+_BCR--ABL1_NALM1_Jaffa     CACAGCATTCCGCTGACCATCAACAAGGAAgaAGCCCTTCAGCGGCCAGTAGCATCTGAC
                                                                 ****************** ********************************

chr3:129574336:-_chr3:129671264:-_PLXND1--TMCC1_HCC1187_Probe     -----CCTGCCCCGCCCCCGAGATCCACGCGATCGAACGGTTGGAAGTAAGCAGCC----
chr3:129574336:-_chr3:129671264:-_PLXND1--TMCC1_HCC1187_Jaffa     TGGCACCTGCCCCGCCCCCGAGATCCGCGCgaTCGAACGGTTGGAAGTAAGCAGCCTTGC
                                                                        ********************* *****************************
```

Fig S4: Alignment of the 2 false negative Cosmic fusion probes with one read mapping this fusion described in the Ligea portal (http://hpc-bioinformatics.cineca.it) with the Jaffa algorithm. Red box: variant close to the fusion junction; green box: fusion junction.

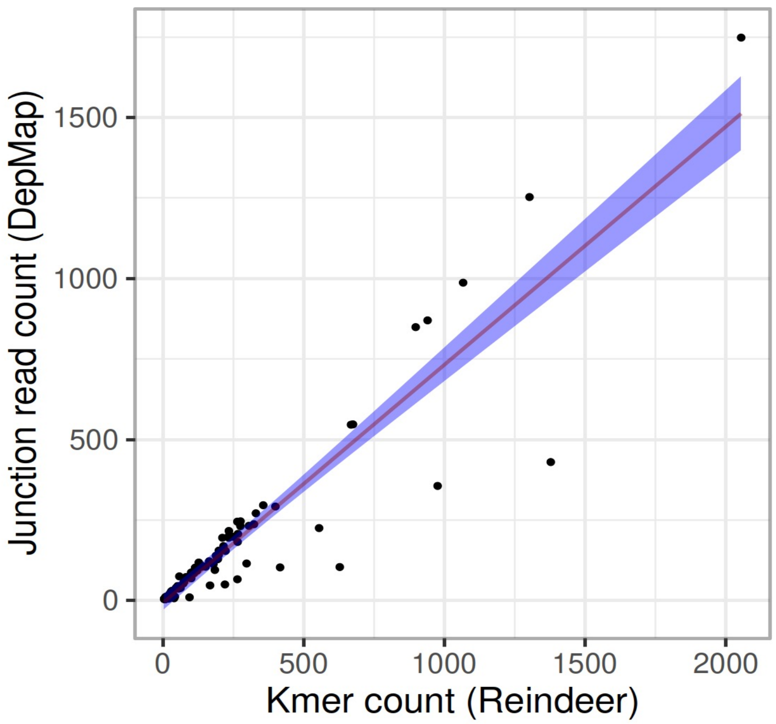

Fig S5: Correlation between Junction read count given by DepMap and Reindeer raw counts for Cosmic fusions.

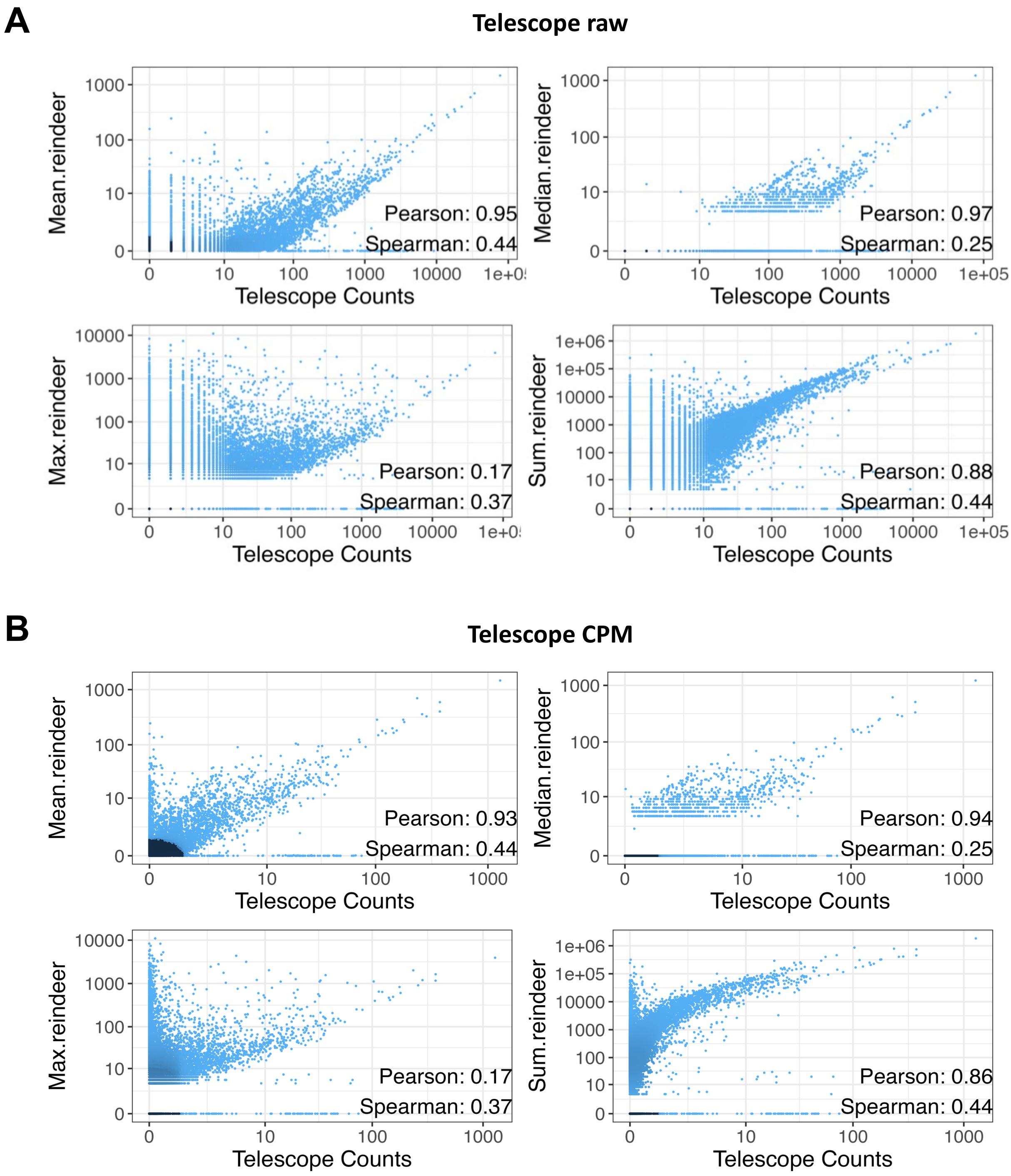

Fig S6. Correlation between Reindeer and Telescope counts. Counts were estimated for 1000 HERV repeats on 56 CCLE colon samples. A: Telescope raw counts. B: Telescope CPM.

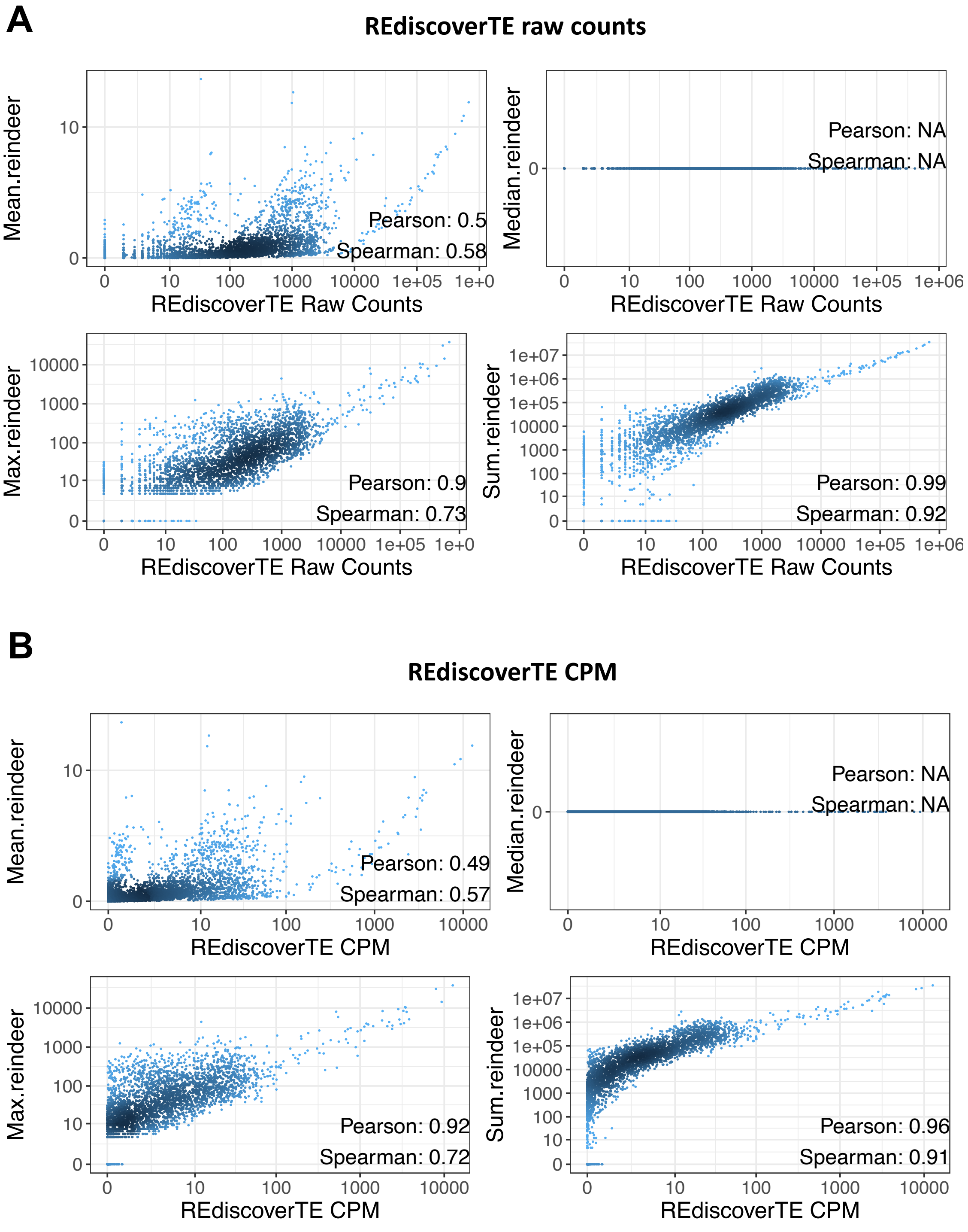

Fig S7: Correlation between Reindeer and REdiscoverTE counts. Counts were estimated for 58 ERV families quantified in 56 CCLE colon samples. **A:** Reindeer vs. REdiscoverTE raw counts. **B:** Reindeer vs. REdiscoverTE CPM. Note that Reindeer median counts for repeat families are always zero. Indeed, each repeat family is composed of hundreds of loci, of which most are silent (count=0), hence the zero median.

A

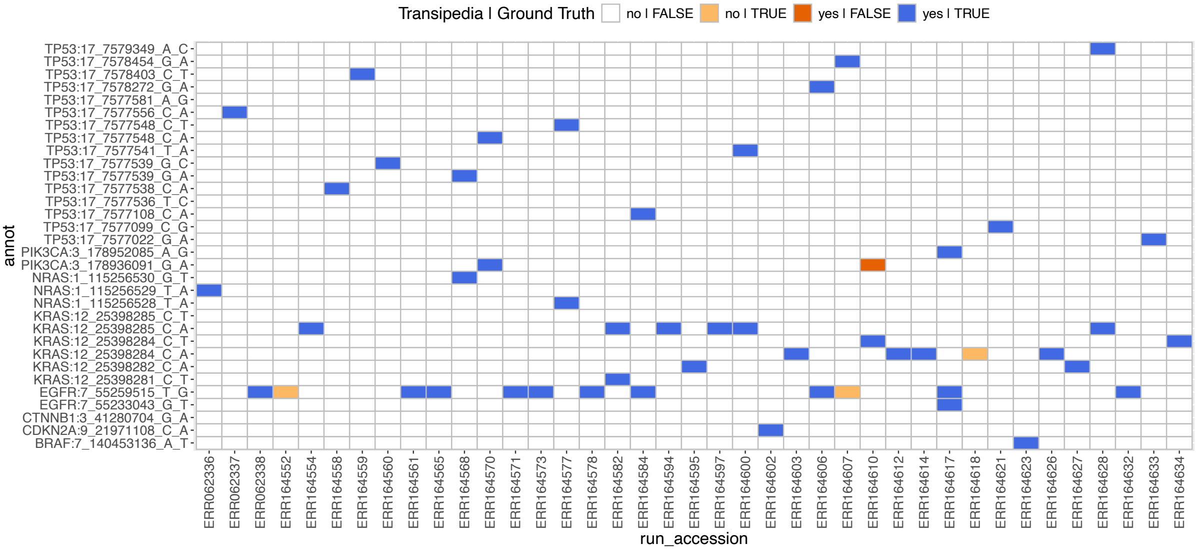

B

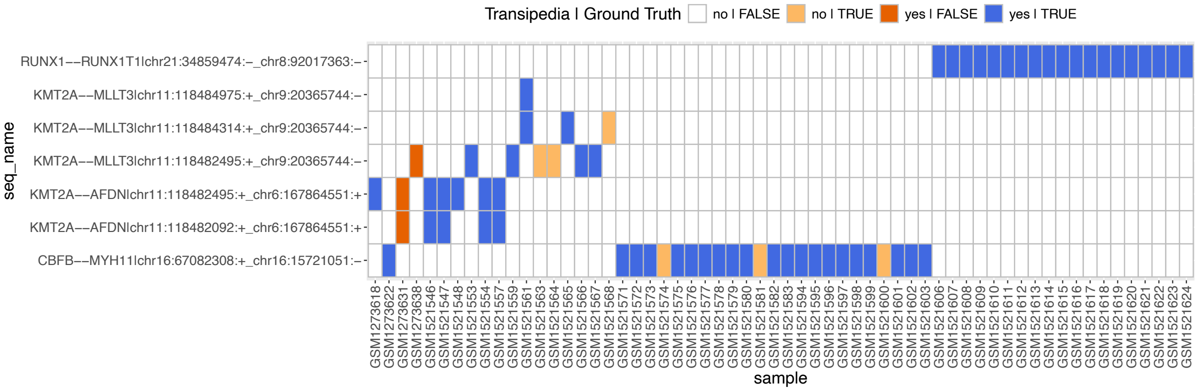

Fig S8: Matches of Depmap-derived probes in independent datasets. A. Mutation probes searched in lung cancer data. **B**. Fusion probes searched in Leucegene data. See dataset references in Methods. Color code: white: true negative, green: false negative, red: false positive, blue: true positive.

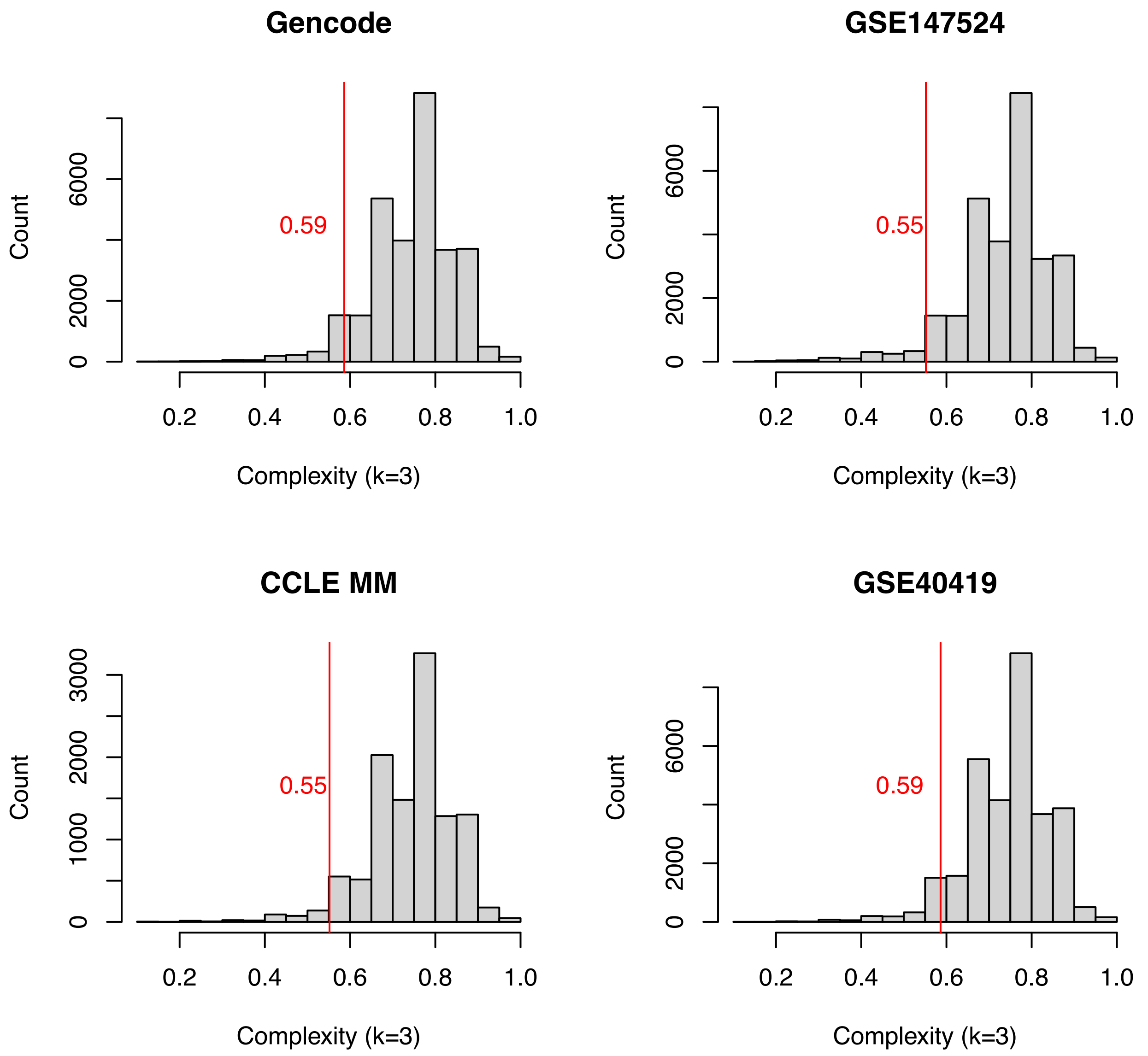

Fig S9: Distribution of 31-mer complexity in four RNA sequence datasets. Complexity is measured for each 31-mer in the dataset as: (number of distinct 3-mers) / (total number of 3-mers). Distribution is shown for Gencode V44, GSE147524 (SRA RNA-seq), GSE40419 (SRA RNA-seq) and CCLE multiple myeloma RNA-seq samples. Fastq datasets were quality-trimmed (Q20) prior to 31-mer extraction. 11,000 to 31,000 31-mers (1/10000th) were sampled from each 31-mer table. A vertical red bar is shown at the 5th percentile of each distribution. Based on these observations, the low-complexity threshold for 31-mers was set at 0.55.

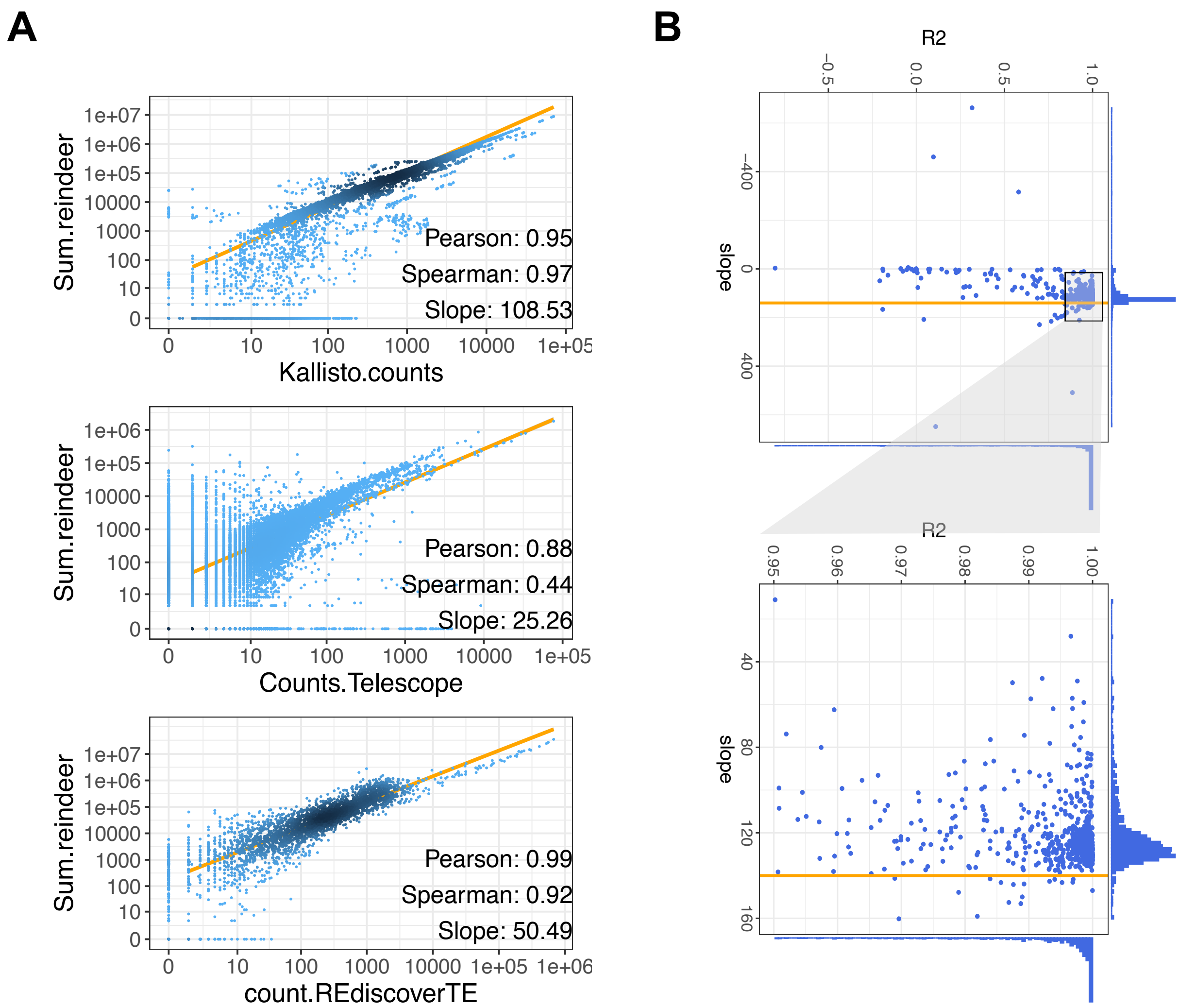

Fig S10: Analysis of correlations between Reindeer sum counts and raw counts from other tools. A. Average fitted lines and slopes for Kallisto, Telescope and REdiscoverTE counts (same data as in Fig S1, S6, S7). **B.** Gene by gene slopes and correlation R^2 for Reindeer vs Kallisto quantificaiton in the MAPQC/SEQC dataset. Each point corresponds to the expression of a single gene measured in 16 conditions. The horizontal line shows a slope of 140. The bottom panel is a zoom on the outlined region on top.
