# Supplementary material for: Transipedia.org: k-mer-based exploration of large RNA sequencing datasets and application to cancer data
Source: Genome Biol. 2024 Oct 10;25:266. doi: 10.1186/s13059-024-03413-5 (PMC11468207; doi:10.1186/s13059-024-03413-5)
Supplement: Supplementary file 3 — Additional file 3: Supplementary Methods. [file 13059_2024_3413_MOESM3_ESM.pdf]

Additional File 3: Supplementary Methods

Transipedia.org: k-mer based exploration of large RNA sequencing datasets and application to cancer data
Bessière et al. 2024

Definitions

k-mer. k-mers are short sequences in a fixed length k. In this article, we limit our scope on 31-mers formed by A, C, G and T, targeting RNA-seq studies.

k-mer count in RNA-seq sequence library. Given a k-mer and an RNA-seq library, the k-mer count is defined as its number of occurrence in the set of sequencing reads.

Sample count vector of a k-mer. Given a k-mer and M ordered RNA-seq libraries, a k-mer's sample count vector is defined as an integer vector in an M-dimensional space, of which the ith component represents the k-mer's count in the ith sequencing library ($i = 1, 2, ..., M$).

Arbitrary sequence. An arbitrary sequence is formed by a finite, variable number of elements selected from a finite set, arranged in an arbitrary order. In the scope of this article, the sequence is considered as RNA sequences, formed by nucleotides A, C, G and T.
In confusion-free context, we shorten the "arbitrary sequence" as "sequence" to be concise.

Sequence query. Given an arbitrary sequence and a series of sequencing libraries, the task of arbitrary sequence query aims to either (i) qualitatively detect its presence/absence, or (ii) quantitatively estimate its expression level, in each of the sequencing library. In this article, we address to the quantification aim, and we suppose the length of the arbitrary sequence is no less than 31 nucleotides (for 31-mers being used).

Component k-mers of a sequence. A sequence of length L can be destructed into $(L - k + 1)$ consecutive k-mers, shifting one nucleotide by step. The set of $(L - k + 1)$ k-mers forms the sequence's component k-mers.

Monotig Given a sequence, a monotig is one of its substring that has a uniform sample count vector across component k-mers.
Note that: (i) a monotig is also an arbitrary sequence *per se*; (ii) unlike k-mers, different monotigs of a same arbitrary sequence do not overlap each other.

Monitigs are the elementary units for Reindeer query. Given a query sequence Q, one its monotig m is determined by a pair of positions $b_Q(m)$ and $e_Q(m)$, respectively meaning the starting and ending k-mers in the sequence Q.

In the context where the query sequence Q is defined, we simplify the writing from $b_Q(m)$ to b_i and $e_Q(m)$ to e_i, where i is the monotig's ordinal number along Q, i.e., determined as between the b_i^{th} and e_i^{th} k-mers along Q.

A monotig of a given query sequence can or cannot be found in one specific library S. A successful query is gained when the monotig has sufficient proportion of component k-mer counts in S to support the hit. The minimum proportion is set by user with -P parameter of the software.

The count of a given monotig m in a library S is reported by a triplet b_i-e_i:q_i, in which b_i-e_i specifies the starting and ending k-mers along the query sequence for m, and q_i indicates the query result of the monotig's component k-mers in library S. In the case of successful query, q_i is returned as an integer, while when the query is failed, it is originally returned as an "*" sign by the software, and we replace the "*" sign by 0 in this article.

When Reindeer queries a sequence composing n monotigs, it returns for each library S a list of triplets indicating monotigs' query results, formed as $b_1 - e_1 : q_1$, $b_2 - e_2 : q_2$, ..., $b_n - e_n : q_n$, where each triplet $b_i - e_i : q_i$ reports the count of the ith monotig ($i = 1, 2, ..., n$).

Conversion from read to k-mer counts

There is a difference in scale between Reindeer sum counts and raw counts from the various quantification tools (Fig 2C,F, Fig. 4A,B). The average correction (slope) is shown in Additional file 1: Fig S10A for each tool. For instance, Kallisto counts are related to Reindeer counts by a factor of 108.53 in average. We found this factor to be related to two phenomena:

- k-mer to fragment or read conversion: given 70 31-mers in each 100-nucleotides read in the CCLE dataset, k-mer counts are expected to be about 140 times higher than fragment counts (each fragment = 2 reads × 70 k-mers),
- query masking: when k-mers in a query are masked, they do not contribute to Reindeer sum counts, whereas other counting methods have no such constraint.

The second point is illustrated by the presence of parallel lines in Additional file 1: Fig S10A, suggesting different correction factors for different genes. This is confirmed in Additional file 1: Fig S10B, showing slopes of Reindeer vs. Kallisto correlations for independent genes. Most genes have highly correlated counts (R^2 near 1) but each with a distinct slope.

In summary, the correction factor for converting Reindeer sum-counts to read counts from Kallisto or other tools depends both on the library (read size and paired-end status) and the ratio of masked k-mers in each query. A future Reindeer query environment could take these parameters into account and automatically adjust raw k-mer counts in a dataset- and query-independent way. This is not straightforward as it requires transmission of metadata from the indexing and masking steps to the query processing step.

Note that we did not intend to consider here another, non linear, factor of variation, namely that Reindeer counts were obtained based on principal transcript while Kallisto counts were obtained using all isoforms.

Implementation details

To enable real-time queries with no index loading overhead, we wrote the *rdeer-service* tool that pre-loads one or several indexes in memory and runs queries (`https://github.com/Bio2M/rdeer-service`) on those indexes. *rdeer-service* uses a metadata file, *fos.txt*, stored in the same directory as the index, containing names and total numbers of k-mers for each sample. This allows displaying sample names in outputs and computing normalized counts, in the form of counts per billion k-mers.

A *fos.txt* file is not produced by Reindeer index but can be created by the *fos_builder* Python script (`https://github.com/Transipedia/fos_builder`). For normalization, *fos_builder* uses information from *multiqc*, thus requiring prior run of *fastqc* and *multiqc* on all samples.
