# Supplementary material for: Transipedia.org: k-mer-based exploration of large RNA sequencing datasets and application to cancer data
Source: Genome Biol. 2024 Oct 10;25:266. doi: 10.1186/s13059-024-03413-5 (PMC11468207; doi:10.1186/s13059-024-03413-5)
Supplement: Supplementary file 4 — Additional file 4: Review History. [file 13059_2024_3413_MOESM4_ESM.docx]

**1^st^ round**

**Reviewer 1**

These results are obtained by an adapted implementation of the existing Reindeer index [8], which includes memory optimizations (e.g. a socket mode and storing part of the index on disk), query engineering (query design, masking, and post-processing, which may also be of interest in other applications). Experiments are conducted on the Cancel Cell Line Encyclopedia, and a ready-made index is also presented for it.

Overall, this paper presents the first practical implementation of a web-server for reference-free quantitative queries in RNA-seq datasets of over a thousand samples, requiring only moderate computational resources (25Gb RAM, 250 Gb SSD storage), and supporting moderately-sized queries in seconds. This can provide a direct access to raw unprocessed RNa-seq data that would otherwise require resource-intensive pipelines.

I consider the methods to be appropriate, with sufficient details provided, and the conclusions adequately supported by the data. Overall, I consider this to be a significant advance over the state of the art, and of broad interest to others in the field.

I have only a few minor comments, as follows:
- Sometimes ">=" is used, sometimes "≥". I would uniformize this notation.
- page 7: "(i) Reindeer index building is a separate action that is computer intensive and best performed by bioinformaticians." Maybe the sentence "and best performed by bioinformaticians" can be replaced by a few technical difficulties that need to be performed by bioinformaticians?
- page 8, under "Updates to Reindeer": maybe you could briefly mention what does the hashmap precisely store, and why itself fits the memory. Is the count matrix the expensive part of Reindeer?
- Caption of Fig S1: "serie" -> "series"

**Reviewer 2**

The authors present a case study for a web service that provides an index of more than 1000 RNA-Seq experiments and associated k-mer counts that can be interactively queried. With the goal to make this kind of (widely used) data more accessible for researchers, the study describes o full-text queries on the k-mer index can be used to retrieve k-mer counts in all source samples and aggregate them in a biologically interpretable manner. The web service is based on the previously published and subsequently adapted Reindeer approach by the same authors. Using all RNA-Seq samples of the Cancer Cell Line Encyclopedia (CCLE) as a testing bed, the authors first evaluate the accuracy of the query results and then showcase different queries of sequence features that usually are not found in the standard annotation, such as gene fusions, somatic mutations, or aberrant splicing junctions. The authors find that the returned counts are accurate and the detection of novel sequence features is sensitive. For increasing specificity of the results a certain amount of "query engineering" is necessary to reduce the rate of false positive results.
Overall, the manuscript is well written and easy to follow. The descriptions are comprehensive and the figures illustrate the main points of the work. The presented service is useful and worth a further extension. The only current limitation of the method is that the minimal query length is restricted to the length of k that has been used to generate the index and that only exact matches are currently recognised. A sensitive alignment of the query to the index is currently not possible. Nevertheless, the presented service seems a great addition to the tool chain for quantitative data exploration.

Overall, I mostly have minor comments (provided in the order of the text):

* Depending on the use case, different steps of query pre-processing need to be completed. For instance, masking all k-mers from the query that are non-unique in the respective genome (e.g., human) is quite a burden on the user, as this has to be re-done for every query. Could it make sense to offer an index that pre-masks these k-mers already?

* A second point on query engineering - The authors describe the discovery of novel biological signal as one of the main use-cases for the service. I would argue that for novelty detection query engineering can be hard as the underlying ground truth is unknown. The only option I see is to engineer the query on a comparable dataset with similar properties (and available ground truth) and then hope that the insights are transferable to the dataset of interest. Could the authors comment on that?

* One page 7 the authors write: "We identified the optimal setting whereby SNPs were almost never a problem while retaining a high specificity." I read the sentence several times, but don't really understand what it means. Could you please clarify?

* On page 9 in the section of "Query preparation" the authors write: "3-mer complexity defined as ((number of distinct 3-mers) / (total number of 3-mers))". The statement leaves open what the total number of 3-mers is computed over. All possible 3-mers? All 3-mers in the current query? All 3-mers in the transcriptome (likely the same as all possible)?

* On page 9 in the section of "Processing of query results" the authors write: "Query abundance in Fig. 2A-C was computed as the mean, median, maximum and sum values of monotig counts." This is a bit misleading, as Figure 2 only shows mean and sum. The statement would make more sense, if also Figure S2 were referenced here.

* On page 9 in the section of "RNA mutations". There seems to be a reference missing for vcf2seq. Also, the cutoff for --max-on-transcriptome seems quite high with 100. Can the authors explain the motivation behind this choice?

* In the caption for Figure 1, the authors write: "For massive query and/or pipelines, the index can be queried from the command line using rdeer-client (rdeer).". Just for clarification. Does the client connect to the transipedia server or will it only connect to a server running locally? This is a bit ambiguous in panel A.

* For the query times summarised in Table 2. Are all queries actually present in the index? Would it make a difference time-wise whether a query is present or not? How is output time considered in these numbers? For instance, it might make a difference to retrieve counts for a query that is only found in one sample vs one that is found in all samples (given that there are thousands of samples).

* In Figure S3 the quantification for mutation and fusion probes is given as the mean over all k-mer counts of the query. Given that the query is (by design) quite short and that SNVs near the fringes of the query will cause some k-mer counts to be 0, wouldn't their effect on the quantification be better controlled via a median rather than a mean?

* One question on the data visualisation of transipedia.fr. Using CCLE as an index and choosing the three given example queries, the heatmap summarises the results per given query. When switching to other plots, such as bar-, box-, or violin-plot, the identity of the queries seems to get lost and it is unclear how the data points are being aggregated.

Typos:
* page 3: mutiple --> multiple
* page 5: repeats impedes --> repeats impede
* page 6: litterature --> literature
* page 10: hostpot --> hotspot
* page 10: Transposable elements quantification --> Transposable element quantification
* page 15: counts in the S --> counts in S

**Authors’ response**

Reviewer #1:

These results are obtained by an adapted implementation of the existing Reindeer index [8], which includes memory optimizations (e.g. a socket mode and storing part of the index on disk), query engineering (query design, masking, and post- processing, which may also be of interest in other applications). Experiments are conducted on the Cancel Cell Line Encyclopedia, and a ready-made index is also presented for it.

Overall, this paper presents the first practical implementation of a web-server for reference-free quantitative queries in RNA-seq datasets of over a thousand samples, requiring only moderate computational resources (25Gb RAM, 250 Gb SSD storage), and supporting moderately-sized queries in seconds. This can provide a direct access to raw unprocessed RNa-seq data that would otherwise require resource-intensive pipelines.

I consider the methods to be appropriate, with sufficient details provided, and the conclusions adequately supported by the data. Overall, I consider this to be a significant advance over the state of the art, and of broad interest to others in the field. I have only a few minor comments, as follows:

- Sometimes ">=" is used, sometimes "≥". I would uniformize this notation.

A: fixed

- page 7: "(i) Reindeer index building is a separate action that is computer intensive and best performed by bioinformaticians." Maybe the sentence "and best performed by bioinformaticians" can be replaced by a few technical difficulties that need to be performed by bioinformaticians?

A: we changed the sentence similarly to what is suggested

- page 8, under "Updates to Reindeer": maybe you could briefly mention what does the hashmap precisely store, and why itself fits the memory. Is the count matrix the expensive part of Reindeer?

A: The hash map stores the k-mer hash and the most expensive part is indeed the count matrix. We rewrote this part as follows: "This shift involves the ability to serialize the count matrix of Reindeer, its most expensive part, onto the disk in a compressed format. Conversely, the hashmap has a reduced footprint thanks to an efficient hash function and co-encoded keys. Consequently, we updated Reindeer to only load the hashmap into RAM in the initial phase and read lines of the count matrix only necessary, markedly reducing the total time required for conducting intensive queries, especially when running on SSD."

- Caption of Fig S1: "serie" -> "series"

A: fixed

Reviewer #2:

The authors present a case study for a web service that provides an index of more than 1000 RNA-Seq experiments and associated k-mer counts that can be interactively queried. With the goal to make this kind of (widely used) data more accessible for researchers, the study describes o full-text queries on the k-mer index can be used to retrieve k-mer counts in all source samples and aggregate them in a biologically interpretable manner. The web service is based on the previously published and subsequently adapted Reindeer approach by the same authors. Using all RNA-Seq samples of the Cancer Cell Line Encyclopedia (CCLE) as a testing bed, the authors first evaluate the accuracy of the query results and then showcase different queries of sequence features that usually are not found in the standard annotation, such as gene fusions, somatic mutations, or aberrant splicing junctions. The authors find that the returned counts are accurate and the detection of novel sequence features is sensitive. For increasing specificity of the results a certain amount of "query engineering" is necessary to reduce the rate of false positive results. Overall, the manuscript is well written and easy to follow. The descriptions are comprehensive and the figures illustrate the main points of the work. The presented service is useful and worth a further extension. The only current limitation of the method is that the minimal query length is restricted to the length of k that has been used to generate the index and that only exact matches are currently recognised. A sensitive alignment of the query to the index is currently not possible. Nevertheless, the presented service seems a great addition to the tool chain for quantitative data exploration.

Overall, I mostly have minor comments (provided in the order of the text):

* Depending on the use case, different steps of query pre-processing need to be completed. For instance, masking all k-mers from the query that are non-unique in the respective genome (e.g., human) is quite a burden on the user, as this has to be re- done for every query. Could it make sense to offer an index that pre-masks these k- mers already?

A: we’d rather not mask repeated k-mers in the index as some applications may involve counting those k-mers (eg. quantifying a given family of repeats containing repeated k-mers that are not found outside this family). However we will try to provide users with as many masked probes as possible. (to be found in https://github.com/Transipedia/Reindeer-use-cases/tree/main/probes)

* A second point on query engineering - The authors describe the discovery of novel biological signal as one of the main use-cases for the service. I would argue that for novelty detection query engineering can be hard as the underlying ground truth is unknown. The only option I see is to engineer the query on a comparable dataset with similar properties (and available ground truth) and then hope that the insights are transferable to the dataset of interest. Could the authors comment on that?

A: To address this, we interrogated two independent datasets with known ground truth (a lung cancer and a leukemia dataset) with mutations and fusion probes designed from the Depmap/CCLE database. Results are presented in a new Result section “Transferring probes across datasets”. Overall we observed that probes built from Depmap had high recall and accuracy in the independent cohorts, however it is important to remmeber that the principle of k-mer indices such as Reindeer is that probe sequences must be given as input and cannot be discovered in the index.

* One page 7 the authors write: "We identified the optimal setting whereby SNPs were almost never a problem while retaining a high specificity." I read the sentence several times, but don't really understand what it means. Could you please clarify?

A: Changed to: "We identified the optimal setting whereby flanking SNPs minimally interfered with mutation calling while maintaining high specificity"

* On page 9 in the section of "Query preparation" the authors write: "3-mer complexity defined as ((number of distinct 3-mers) / (total number of 3-mers))". The statement leaves open what the total number of 3-mers is computed over. All possible 3-mers? All 3-mers in the current query? All 3-mers in the transcriptome (likely the same as all possible)?

A: this was the number of 3-mers in each k-mer. Now specified.

* On page 9 in the section of "Processing of query results" the authors write: "Query abundance in Fig. 2A-C was computed as the mean, median, maximum and sum values of monotig counts." This is a bit misleading, as Figure 2 only shows mean and sum. The statement would make more sense, if also Figure S2 were referenced here.

A: Reference to Fig S2 has been inserted.

* On page 9 in the section of "RNA mutations". There seems to be a reference missing for vcf2seq. Also, the cutoff for --max-on-transcriptome seems quite high with 100. Can the authors explain the motivation behind this choice?

A: vcf2seq is a custom script available on github. We now cite the github URL.

* In the caption for Figure 1, the authors write: "For massive query and/or pipelines, the index can be queried from the command line using rdeer-client (rdeer).". Just for clarification. Does the client connect to the transipedia server or will it only connect to a server running locally? This is a bit ambiguous in panel A.

A: Massive queries have to be performed on a local server. Now clarified in the figure legend.

* For the query times summarised in Table 2. Are all queries actually present in the index? Would it make a difference time-wise whether a query is present or not? How is output time considered in these numbers? For instance, it might make a difference to retrieve counts for a query that is only found in one sample vs one that is found in all samples (given that there are thousands of samples).

A: The number of samples with zero counts does not impact query time (provided the number of samples remains the same), however a sequence that is altogether absent in the index would result in the fastest query time. Queries in Table 2 are performed with common mRNAs whose k-mers are mostly present in the CCLE dataset, thus query times are rather a worst case scenario.

* In Figure S3 the quantification for mutation and fusion probes is given as the mean over all k-mer counts of the query. Given that the query is (by design) quite short and that SNVs near the fringes of the query will cause some k-mer counts to be 0, wouldn't their effect on the quantification be better controlled via a median rather than a mean?

A: Actually figure S3 says “mean of positive counts”, thus zeros are not counted.

* One question on the data visualisation of transipedia.fr. Using CCLE as an index and choosing the three given example queries, the heatmap summarises the results per given query. When switching to other plots, such as bar-, box-, or violin-plot, the identity of the queries seems to get lost and it is unclear how the data points are being aggregated.

A: In the boxplot or violin plot modes, it is possible to select one or more samples by double clicking on them and visualize all queries for these samples. However the barplot mode only shows the sum of counts for all queries. We are trying to make this as intuitive as possible. We will rely on user feedback to improve this after publication.

Typos:

* page 3: mutiple --> multiple

* page 5: repeats impedes --> repeats impede

* page 6: litterature --> literature

* page 10: hostpot --> hotspot

* page 10: Transposable elements quantification --> Transposable element quantification

* page 15: counts in the S --> counts in S

A: all fixed.
